# Mechanistic Study of the Kinetic Phenomena Influencing the Bacteriostatic Action of Silver Ions in Agar Bioassays

**DOI:** 10.3390/antibiotics10040368

**Published:** 2021-03-31

**Authors:** Louis Cornette de Saint Cyr, Guillaume Ramadier, Azariel Ruiz Valencia, Jean-Pierre Méricq, Laurence Soussan

**Affiliations:** 1 Institut Européen des Membranes, IEM–UMR 5635, University Montpellier, ENSCM, CNRS, 34090 Montpellier, France; louis.cornette-de-saint-cyr@umontpellier.fr (L.C.d.S.C.); azarv86@hotmail.com (A.R.V.); jean-pierre.mericq@umontpellier.fr (J.-P.M.); 2 Département de Physique, École Normale Supérieure de Lyon, 69364 Lyon, France; ramadier.guillaume@icloud.com

**Keywords:** bacteriostatic studies, kinetics, experiment and model, agar diffusion test, diffusion, bacterial growth, antibacterial action, bacterial inhibition, silver ions, *E. coli*

## Abstract

Bacteriostatic action of a biocidal agent results from the cumulative impact of different kinetics, including those of bacterial growth, mass transfer of the agent and its antibacterial action against the targeted bacteria. Current studies on bacteriostatic effects always directly consider the combination of these kinetics at given times, without discrimination between each other. This work introduces a novel approach, consisting of first studying independently, by the experiment and the model, the different kinetics involved, and then in coupling these kinetics to obtain a model that will be confronted with experimental data. An agar diffusion test with silver ions against *Escherichia coli* bacteria was implemented herein to assess the relevance of this approach. This work achieved to characterize the different kinetics and to propose a dynamic model combining them, which fits the experimental data with a silver diffusivity in the biofilm fixed to 7.0 ± 0.1 × 10^−12^ m^2^ s^−1^. This study also proves that the diffusive phenomenon was limiting the bacteriostatic action of silver ions over the test duration.

## 1. Introduction

Bacteriostatic action, i.e., the inhibition of bacterial growth, is useful, and required in numerous application fields, such as drug delivery to prevent tissue infections (e.g., antibacterial plasters), inhibition of biofilm formation on food packaging, or on membranes used for water and air treatments [1,2,3,4,5,6,7]. The bacteriostatic efficiency depends on several factors, such as the antibacterial agent used, its concentration, the bacterial strains, the bacterial concentration involved, and the kinetics (i.e., transport of the bacteriostatic agent, bacterial growth and antibacterial action), resulting in a certain contact time between bacteria and the antibacterial agent [8]. As an example, a too slow transport of the biocidal agent may not prevent bacteria from growing since bacteria can multiply faster than they die. On the contrary, a too fast transport can induce an excessive consumption of biocidal agent and expose the receiving environment to high concentrations that can be possibly harmful [9,10]. Thus, it is very important to determine kinetics of antibacterial agent transfer, bacterial growth, and antibacterial action.

In this study, a usual and visual agar diffusion test was chosen to illustrate a bacteriostatic system where kinetics characterizations could be done. Silver ions (Ag^+^) are a well-known biocidal agent [11,12] and were so selected for this test. Silver ions have indeed powerful antibacterial properties that were demonstrated and characterized by numerous studies [1,2,13,14,15,16,17,18,19]. In particular, Ag^+^ is able to (i) interact with the thiol groups (-SH) of the membrane cell proteins to form S-Ag bonds, leading to the inhibition of the biologic activity of these proteins (it is notably the case of the respiratory chain proteins), and (ii) induce protein agglomeration around DNA as a defense mechanism when it has penetrated in the cell, thus inhibiting DNA replication [1,2,13,14,15,16,17,18,19]. These processes could lead to cell death. Herein, silver ions were deposited in a well, made in the center of a nutritive agar plate that was priorly inoculated on its surface by a non-pathogenic *Escherichia coli* bacterium exhibiting similar shapes and biochemical properties as pathogenic *E. coli*. In this configuration, different coupled kinetics occur: bacterial growth on the nutritive agar (inducing a biofilm formation), Ag^+^ diffusion in the agar and in the biofilm formed and antibacterial action of the diffused Ag^+^. Under certain conditions, the cumulative effect of these kinetics can result in the formation of inhibition halos (or inhibition zones) around the silver well that are enlarged over time due to silver diffusion. These inhibition halos are zones where bacteria were prevented from growing on the agar by action of silver. They correspond to agar solely, by opposition to the colonized agar where a bacterial biofilm has formed.

To the best of our knowledge, the modeling currently used to assess the evolution of the inhibition zones radii is based on a diffusive model (Fick’s law) traducing the antibacterial agent diffusion within the agar and on the experimental determinations of (i) the Minimum Inhibitory Concentration (MIC), i.e., the minimal concentration of antibacterial agent for which an inhibition zone appears after a given incubation period of the agar plates (usually 24 h or 48 h), and (ii) the critical time (T_c_) that is the incubation time from which the bacterial cells become more numerous than what the MIC can inhibit [20,21,22]. However, the MIC already couples the different kinetics and the diffusion approach does not take into account the bacterial growth that can impact the diffusive transport [20,21,22]. This work aimed to distinguish experimentally and by the model the different kinetics (diffusion vs. bacterial growth and biocidal action) to get an overview of the system dynamics and identify the limiting phenomenon over the test duration. Thus, in this study, experiments were first designed to study independently the kinetics of silver diffusion, bacterial growth, and antibacterial action of silver. In addition, kinetics models were implemented. The correlation between experiments and models allowed notably to (i) obtain Ag^+^ diffusivities (in agar but overall in the biofilm formed) and the kinetic constants for bacterial growth and Ag^+^ biocidal action; (ii) identify the limiting phenomenon over time; and finally (iii) propose an innovative model combining all the kinetics that allows to simulate the coupling as a function of time and space. More generally, such an approach could be also envisaged to obtain models describing other bacteriostatic systems that involve diffusive materials.

## 2. Results and Discussion

Pure diffusion, pure bacterial growth, and pure antibacterial action of silver ions will be first decoupled and characterized by both experience and model to propose consistent kinetic models for each of this phenomenon. Then, these kinetics will be combined in a global model that will be compared to experimental data (i.e., inhibition zones radii).

### 2.1. Pure Diffusion of Silver Ions in the Agar

#### 2.1.1. Modeling the Concentration Profile of Ag^+^ Diffusing in the Agar Plate

The diffusion of the antibacterial agent (Ag^+^) in the agar exempt of bacteria was first studied in order to test the reliability for this specific application of both the COMSOL module used and the experiments carried out. Agar can be considered as a continuous and homogenous static medium, whose dynamic viscosity was fixed to 2.2 Pa s [23] and density to 0.799 g mL^−1^ (obtained in this study by weighting a known agar volume). Thus, diffusion transport follows the Fick’s Laws [20] and the flow density J_Ag_^+^ of Ag^+^ diffusing in the agar medium was modeled by the first Fick’s Law (Equation (1)):

(1) $$\vec{J_{{\mathrm{Ag}}^{+}}}=-D_{{\mathrm{Ag}}^{+}}\vec{\nabla }\left(C_{{\mathrm{Ag}}^{+}}\left(\;\vec{r},t\right)\right)$$

where:

J_Ag_^+^ (mol m^−2^ s^−1^) is the flow density of Ag^+^ diffusing in the agar medium;
C_Ag_^+^ (mol m^−3^) is the concentration of Ag^+^ at a position r and at a time t;
D_Ag_^+^ (m^2^ s^−1^) is the diffusivity of Ag^+^ in the agar medium.

Diffusion is a microscopic phenomenon and a microscopic mass balance carried out on a portion of agar plate in mole of Ag^+^ per second gives the second Fick’s Law (Equation (2)):

(2) $$\frac{\partial C_{{\mathrm{Ag}}^{+}}\left(\vec{r},t\right)}{\partial t}=-\vec{\nabla }{\;J}_{{\mathrm{Ag}}^{+}}$$

By replacing the expression of the flow density$\;\vec{j_{{\mathrm{Ag}}^{+}}}$ in Equation (2), Equation (3) comes:

(3) $$\frac{\partial C_{{\mathrm{Ag}}^{+}}\left(\vec{r},t\right)}{\partial t}=D_{{\mathrm{Ag}}^{+}}\vec{\nabla }{\;}^{2}C_{{\mathrm{Ag}}^{+}}\left(\vec{r},t\right)$$

**Figure 1:**
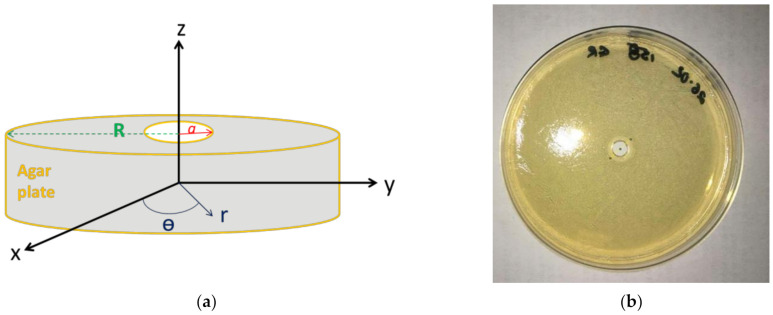
Agar plate with a well in the center: (**a**) scheme and (**b**) top view of the plate.

In agreement with the experimental system, a cylindrical geometry of the agar plate was considered (Figure 1) with a cylindrical coordinate system. We looked for a C_Ag_^+^ (r, t) solution that has to satisfy the initial conditions (IC) and boundary conditions (BC) given by Equations (4) and (5) respectively:

(4) $$\mathrm{IC}:\;C_{{\mathrm{Ag}}^{+}}\left(r\leq a,0\right)\;=C_{{\mathrm{Ag}}_{0}^{+}}{\;\mathrm{and}\;C}_{{\mathrm{Ag}}^{+}}\left(r>a,0\right)\;=0$$

where:

a (m) is the radius of the well made in the agar plate center (Figure 1) and C_Ag0_^+^ (mol m^−3^) the initial concentration of the Ag^+^ solution that was deposited in the well

(5) $$\mathrm{BC}:\;\forall \;t\in R_{+},\;C_{{\mathrm{Ag}}^{+}}\left(r=R,t\right)=0$$

where R (m) is the radius of the agar plate (Figure 1)

This first model was implemented in the COMSOL mass transfer module. From the modeling of Ag^+^ concentration profile, a modeling of halo radius was determined by considering the halo as the zone where the Ag^+^ concentration was different from zero.

#### 2.1.2. Experiments: Measuring the Radius of the Diffusion Halos over Time

Experiments were carried out as described in Section 3.3.1. Silver solution was stained with a red food dye before being deposited in the well of the agar plate that was not seeded; the concentration of silver ions was fixed to 100 mg L^−1^. Then the formation and progression of the dyed halos around the wells were monitored over time. The radius of the dyed halos corresponds to the diffusion radius since diffusion was the only phenomenon occurring in the experiment.

**Figure 2:**
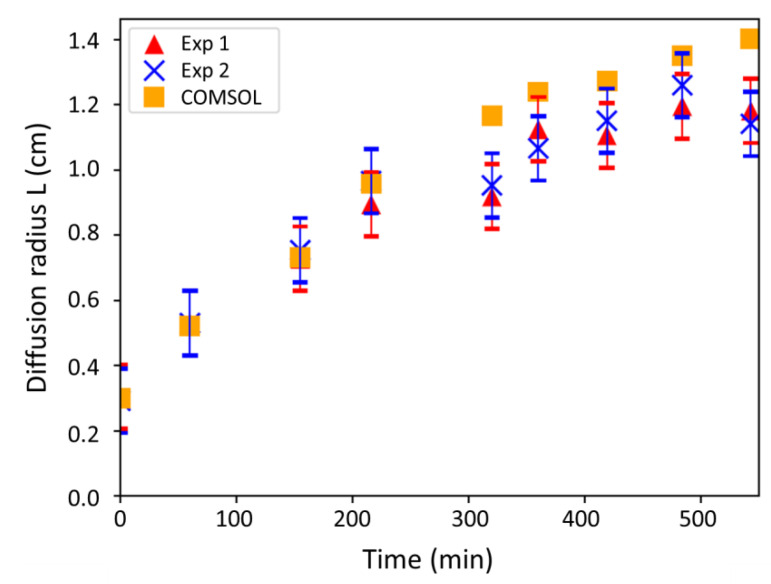
Comparison of the diffusion radii obtained by the model and the experiments: results over time.

Figure 2 shows the results obtained for the two experiment replicates (Experiment 1 (Exp 1) and Experiment (Exp 2), respectively). Diffusion radii (L) were measured on plate pictures by using the ImageJ software; images illustrating the evolution of the dyed halos over time are given in the supplementary data (Figure S1). Initially, the radius of the halo is equal to the well radius (a).

The uncertainty on the measures was determined by summing three sources of experimental errors on: (i) the graduations of the measuring ruler (Figure S1); (ii) the delimitation between the dyed halo and the agar (Figure S1); and (iii) the distance and residual angle of the camera from the agar plate plane. The calculation of the standard deviation on the ruler graduations is based on the smallest measurable distance (i.e., 0.03 cm) while the other deviations were assessed by Student tests. For the Student tests, the size of the dyed halos were measured twenty times on the first hand, and on the second hand, the size of an object whose the real size is known (for example the ruler) was measured by Image J. An uncertainty of 0.09 cm was obtained.

#### 2.1.3. Comparison between the Model and Experiments

The comparison between the model and experiments is intended to determine the D_Ag_^+^ value, which appears in Equation (3), i.e., the diffusivity of silver ions in Lysogeny Broth (LB) agar (at 15 g_agar_ L^−1^ and 37 °C). The radii (L) of the diffusion halos measured experimentally and obtained by the model were reported against time in Figure 2. For the model, a similar silver concentration as in the experiments was used (i.e., 100 mg L^−1^) and the silver diffusivity D_Ag_^+^ in the agar was fixed to 2.7 × 10^−9^ m^2^ s^−1^ in order to fit the model with the experiment.

With the fitted value of silver diffusivity, Figure 2 shows a quite good correlation between both experimental and modeling values. In addition, the D_Ag_^+^ value of 2.7 × 10^−9^ m^2^ s^−1^ is in accordance with the magnitude order of values reported in the literature for silver ions diffusion in agar gel, i.e., about 10^−9^ m^2^ s^−1^ [24]. This result confirms the reliability of the COMSOL mass transfer module for this study. However, staining the silver solution might have induced chemical composition changes that are likely to interfere with silver diffusion, and could explain some relative deviations observed from 300 min (Figure 2). Staining could obviously interact even more with bacterial growth and biocidal action. Such an approach, thus, appears to be not rigorous enough to conclude on the diffusion phenomenon when it will be coupled with bacterial growth and silver action. This justifies why no staining of the silver solution will be done to determine experimentally the inhibition zones. Nevertheless, the same COMSOL mass transfer module will be used to simulate the diffusion kinetics when bacterial growth and biocidal kinetics will also be considered. The coupling is expected to allow the determination of the silver diffusivity D_Ag_^+^_biofilm_ in the bacterial biofilm formed.

### 2.2. Pure Bacterial Growth

#### 2.2.1. Model

According to Equation (6), the flow of bacteria produced can be defined as the growth rate μ (min^−1^) multiplied by the bacterial concentration N, where the growth rate is a specific rate. The bacterial concentration N is usually expressed in colonies forming units (CFU) mL^−1^, but it can also be expressed in g_dry cell_ L^−1^ or in mol L^−1^. Indeed, the number of bacteria is directly proportional to the mass of dried bacterial cells and the mass can be converted to a mole number since *E. coli* bacteria exhibit a known molecular weight (22 ± 12 kDa, [25]).

(6) $$\frac{\mathrm{dN}}{\mathrm{dt}}=\mu N$$

The growth rate varies with time and usually depends on the concentration of the substrate S in the culture medium limiting the bacterial growth. In this case, the growth rate adopts the Monod law described by Equation (7) [26]:

(7) $$\mu =\frac{\mu_{\max}\left[S\right]}{K_{M}+\left[S\right]}$$

where:

μ (min^−1^) is the growth rate;
μ_max_ (min^−1^) is the maximal growth rate;
[S] (mol L^−1^) is the concentration of the limiting substrate S;
K_M_ (mol L^−1^) is the Monod constant. K_M_ is the concentration of the substrate S for which the growth rate μ is equal to μ_max_/2. Thus, the more K_M_ is small and the more the affinity of the bacteria for the substrate S is important.

When the concentration [S] of the limiting substrate is in large excess compared to K_M_, it comes that the growth rate is maximal (μ = μ_max_). At the opposite, the growth rate is proportional to [S].

#### 2.2.2. Experiments

Experiments were carried out as detailed in Section 3.3.2. The optical density at 600 nm (OD_600_) of the bacterial culture was measured over time during the exponential growth phase. Table 1 shows the results obtained for four independent experiments.

#### 2.2.3. Comparison between the Model and the Experiments

According to Section 3.3.2, the growth rate of the *E. coli* culture was measured in the exponential phase on a rich culture medium (i.e., LB). Thus, in the LB medium, all nutrient substrates were in large excess. In addition, oxygen (O_2_) was continuously provided by air, as was the case for the growth on LB plates. In these conditions, the growth rate was considered maximal (μ_max_). Consequently, Equation (6) becomes Equation (8):

(8) $$\frac{\mathrm{dN}}{\mathrm{dt}}=\mu_{\max}N$$

which gives after integration:

(9) $$\ln\left(N\right)=\mu_{\max\;}\;t+\ln\left(N_{0}\right)$$

where

N_0_ (CFU mL^−1^ or g_dry cell_ L^−1^ or mol L^−1^) is the initial bacterial concentration;
N (CFU mL^−1^ or g_dry cell_ L^−1^ or mol L^−1^) is the bacterial concentration at a time t.

In a preliminary work carried out at the Lab, a linear correlation between the bacterial concentration and OD_600_ was established during the exponential phase (Equation (10)):

N (g L^−1^) = 3.1 × OD_600_ (-) (10)

Equation (9) can thus also be written as describes in Equation (11):

(11) $$\ln\left({\mathrm{OD}}_{600}\right)=\mu_{\max}\;t+\ln\left({\mathrm{OD}}_{600_{i}}\right)$$

where

OD_600i_ (-) is the initial optical density at 600 nm of the bacterial culture;
OD_600_ (-) is the optical density at 600 nm of the bacterial culture at a time t.

**Figure 3:**
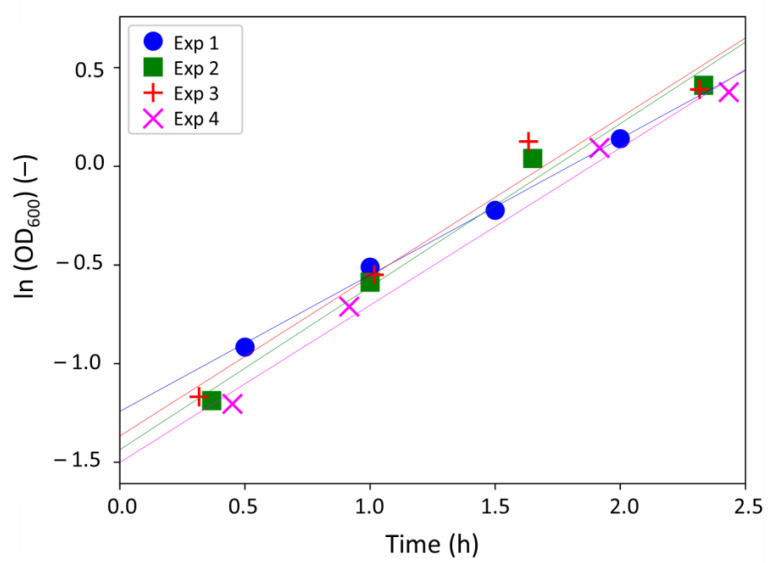
Evolution of ln (OD_600_) with time during the exponential growth phase, for four independent experiments.

Figure 3 plots the experimental results obtained.

As predicted by the model, the evolution of ln (OD_600_) is linear with time, with regression coefficients R^2^ ranging from 0.9684 to 0.9961 (Figure 3 and Table 2) and consistent OD_600i_ values. The experimental data were thus used to determine the maximal growth rate µ_max_.

Replicates were reproducible and the mean value of µ_max_ was fixed to 0.78 ± 0.06 h^−1^ in the rest of the study.

### 2.3. Pure Antibacterial Action of Silver Ions

#### 2.3.1. Modeling the Kinetics of Silver Antibacterial Action

The reaction between bacteria and silver ions can be schematized as follows:

(12) $${\mathrm{Ag}}_{\left(\mathrm{aq}\right)}^{+}+E.coli|⇆|E.coli,{\mathrm{Ag}}^{+}$$

Equation (12) traduces that silver remains linked to the bacterial cell (when agglomerated around DNA) or linked to its cellular compounds, due to a redox reaction between Ag^+^ and the proteins thiol groups (-SH) [14,15,16,17,18]. The most widespread kinetic law to describe the biocidal action of an antibacterial agent is the Chick–Watson law [27]. For silver ions, the flow of inactivated bacteria given by the phenomenological law of Chick–Watson adopts the following form:

(13) $$\frac{\mathrm{dN}}{\mathrm{dt}}=-{k\;C}_{{\mathrm{Ag}}_{0}^{+}}^{n}N$$

where

C_Ag0^+^_ (mol L^−1^) is the initial concentration of silver ions;
N (mol L^−1^) is the bacterial concentration at a time t;
n (-) is an order to determine that is called the dilution coefficient [28];
k is a kinetic constant whose units depends on n

Equation (13) can be integrated and gives:

(14) $$\;\log\left(\frac{N}{N_{0}}\right)=-\frac{k}{\ln10}C_{{\mathrm{Ag}}_{0}^{+}}^{n}\;t$$

where

N_0_ (CFU mL^−1^ or g_dry cell_ L^−1^ or mol L^−1^) is the initial bacterial concentration;
N (CFU mL^−1^ or g_dry cell_ L^−1^ or mol L^−1^) is the bacterial concentration at a time t;
log(N/N_0_) (-) is the bacterial log-removal value. A log-removal value of −1 means that 90 % of the bacteria were removed, while a log-removal value of −2 means that 99% of the bacteria were inactivated, and so on. In the particular case of a total removal (N = 0 CFU mL^−1^), a value of –log(N_0_) was attributed.

In the case of silver ions, the dilution coefficient n was reported to be 1.038, i.e., close to 1 [27]. However, the Chick–Watson law does not take into account the time dependence of the silver concentration C_Ag_^+^ due to the biocidal action of Ag^+^ ions. Indeed, silver ions might be quenched when they are fixed to cellular thiol groups (by the formation of SH–Ag bonds) or confined in the bacterial cell (Equation (12)), leading to a decrease of free Ag^+^ ions in solution available to induce a biocidal action. This explains why the linear model of Chick–Watson is commonly not used for silver ions. The Hom model and the Intrinsic Quenching (IQ) model were therefore developed with an exponential decrease of the silver concentration with time [27] according to Equation (15):

(15) $$C_{{\mathrm{Ag}}^{+}}\left(t\right)=C_{{\mathrm{Ag}}_{0}^{+}}e^{-k_{2}t}$$

with k_2_ (min^−1^) a kinetic constant traducing the quenching of Ag^+^ ions.

In the particular case of Ag^+^ provided by silver nitrates in water at an initial concentration C_Ag0_^+^ of 3.0 mg L^−1^ (corresponding to the experimental concentration), the kinetic laws are respectively [27]:

(16) $$\mathrm{Hom}\;\mathrm{law}:\;\log\left(\frac{N}{N_{0}}\right)=-\;10^{-0.0292}C_{{\mathrm{Ag}}_{0}^{+}}^{1.142}|t^{0.148}\;$$

(17) $$\mathrm{Intrinsic}\;\mathrm{Quenching}\;(\mathrm{IQ})\;\mathrm{law}:\;\log\left(\frac{N}{N_{0}}\right)=\frac{10^{-0.24033}}{0.378}\left[1-e^{-0.378\;t}\right]$$

These models were designed for low silver concentrations (from 0.25 to 2 mg L^−1^) against the Gram-positive *Staphylococcus aureus* bacterium. In a first approach, these laws could be extended to Gram negative bacteria, such as *E. coli,* since studies reported that the action mechanism of silver ions is similar for both Gram bacteria [13,29,30].

#### 2.3.2. Experiments

**Figure 4:**
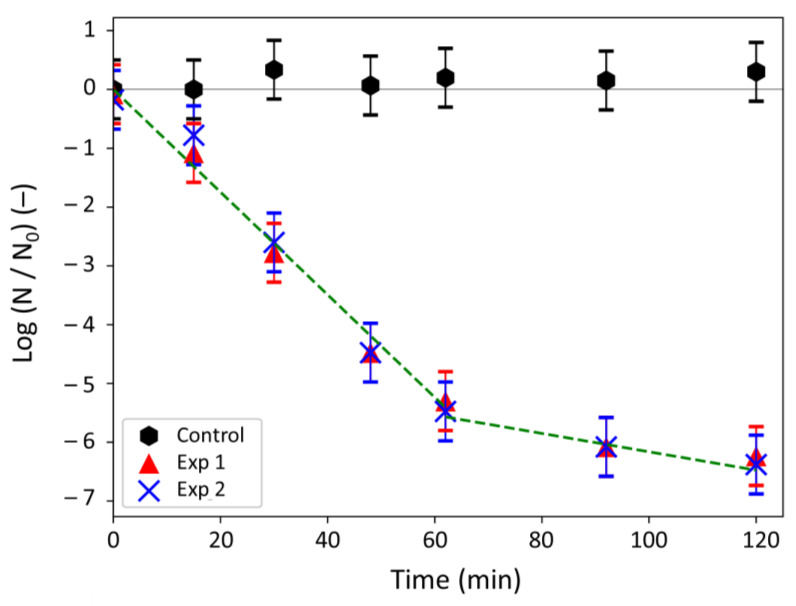
Log-removal values measured over time for silver ions against *E. coli* bacteria. N_0_ refers to the initial bacterial concentration and N to the bacterial concentration at a given time.

The goal of the experiments was to determine the kinetics of the silver ions antibacterial action in a continuous stirred tank reactor whose liquid medium was exempt of nutriments. In this configuration, bacterial growth was avoided and the constant agitation allowed the contact between Ag^+^ ions and bacteria by convection movements of the liquid. The mass transfer of silver toward bacteria was thus expected to be not limiting, which, thus, should induce a limitation by the biocidal phenomenon. Due to the high antibacterial efficiency of Ag^+^ ions [1], a small concentration of silver (i.e., 3.0 mg L^−1^) was chosen for the experiments to permit (i) the monitoring of the bacterial concentration decrease over time, and (ii) the further use of the models presented in Section 2.3.1. The bacterial concentration N was measured over time (Table 3) and the log-removal values were plotted against time in Figure 4.

Table 3 and Figure 4 put in evidence a clear action of the silver ions against bacteria. Indeed, the bacterial concentration remained quasi-constant in the control reactor (i.e., 1.1 ± 0.4 × 10^9^ CFU mL^−1^ on average), whereas it was reduced by 6 log (i.e., by 99.9999%) after 120 min when Ag^+^ ions were present.

Figure 4 evidences that the log-removal decreased quickly over the first 62 min and that the values were proportional to time (R^2^ = 0.9815). Then, the variations of the log-removal values with time became less significant but still adopted a linear behavior (R^2^ = 0.9713). A comparison between the experiments and the different models is presented in the next section.

#### 2.3.3. Comparison between the Models and Experiments

**Figure 5:**
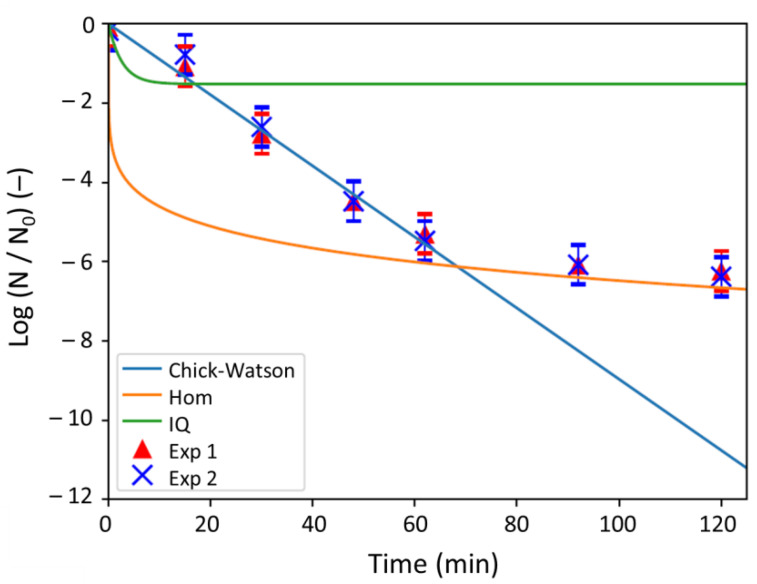
Kinetics of *E. coli* bacterial removal by Ag^+^ ions, obtained by the experiments (Exp 1 and Exp 2, respectively) and the models (Chick–Watson, Hom, and IQ).

Figure 5 shows the log-removal values obtained by the experiments on one hand and on the other hand by the three models (i.e., Chick–Watson, Hom and Intrinsic Quenching ones). It clearly appears that the linear Chick–Watson model (Equation (14)) could suit the experiment data over the first hour but do not describe the whole experiment. As expected, the phenomenological Chick–Watson law is, thus, not adapted in the case of antibacterial action of silver ions. For the Hom model and the Intrinsic Quenching model (IQ), Equations (16) and (17) were plotted. For the Chick–Watson model (Equation (14)), the dilution coefficient n was taken equal to 1.038 [27] and the kinetic constant k (k = 6.6 × 10^−2^ min L mol^−1^) was determined in this study by fitting the experimental data over the first 62 min (Figure 5).

The Chick–Watson model is thus consistent with the experiment beginning only (Figure 5). The Hom model is attractive since the latest log-removal values obtained matches the measured ones (at 92 and 120 min respectively, with log–removal values of about 6, Figure 5). However, the behavior of the Hom model at the beginning of the kinetics (i.e., up to 62 min) is not in agreement with the experiments. Finally, the Intrinsic Quenching (IQ) model seems to be relevant only with the measures over the first 15 min and then adopts a profile completely different (Figure 5). As a conclusion, none of the models reported in the literature for the antibacterial action of Ag^+^ ions are satisfactory enough to describe the experimental results.

Paying attention to the kinetic profile of the log-removal values measured (Figure 4), two affine regressions are distinguishable: one from 0 to 62 min and another from 62 to 120 min. This profile is quite similar to the one observed for chlorine in wastewaters for which the model of Haas and Karra was proposed [31]. Like the Hom and IQ laws, this model takes also into account that the biocidal agent (chlorine or silver ions) can be quenched during the biocidal process, leading to a decrease of its concentration during time. In that case, the quenching of the Ag^+^ ions is done at two different rates (linked to the two linear regressions) and the silver concentration in the reaction medium can be described by Equation (18) [32]:

(18) $$C_{{\mathrm{Ag}}^{+}}\left(t\right)=C_{{\mathrm{Ag}}_{0}^{+}}\left({\mathrm{Xe}}^{-k_{1}t}+\left(1-X\right)e^{-k_{2}t}\right)$$

where

k_1_ and k_2_ (min^−1^) are general kinetic constants (k_1_ = 1 min^−1^ and k_2_ = 0.003 min^−1^);
X (-) is an empirical constant (X = 0.9).

**Figure 6:**
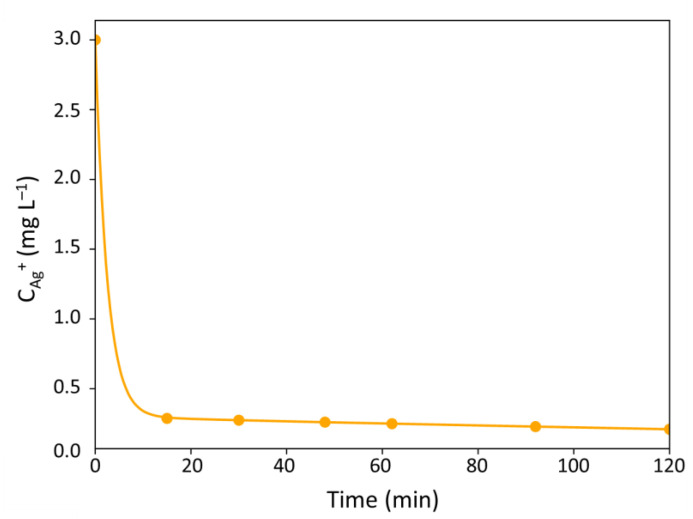
Haas and Karra model: concentration profile of Ag^+^ ions with time.

The kinetic modeling of the Ag^+^ concentration in the reaction medium is thus given in Figure 6:

Based on this curve (Figure 6), the bacterial removal can be divided in three zones:

- First zone: Ag^+^ ions are fully available (C_Ag_^+^ ≃ C_Ag0_^+^) and the flow of inactivated bacteria is maximum:

(19) $$\frac{\mathrm{dN}}{\mathrm{dt}}=-k|C_{{\mathrm{Ag}}_{0}^{+}}N$$

- Second zone: an intermediate and quick zone where free and quenched Ag^+^ ions coexist in the reaction medium; the concentration C_Ag_^+^ depends on time according to Equation (18),
- Third zone: the Ag^+^ concentration is strongly reduced and remains constant at C_Ag_^+^ ≃ 0.1 C_Ag0_^+^ ≃ 0.3 mg L^−1^:

(20) $$\frac{\mathrm{dN}}{\mathrm{dt}}=-k^{\prime \prime }C_{{\mathrm{Ag}}^{+}}N=-0.1k^{\prime \prime }C_{{\mathrm{Ag}}_{0}^{+}}N=-k^{\prime }C_{{\mathrm{Ag}}_{0}^{+}}N$$

with k’ = 0.1 k’’

By integrating the Equations (19) and (20), two linear laws are obtained:

(21) $$\log\left(\frac{N}{N_{0}}\right)=-\frac{k|C_{{\mathrm{Ag}}_{0}^{+}}}{\ln\left(10\right)}t\;\mathrm{for}\;C_{{\mathrm{Ag}}^{+}}\leq C_{\mathrm{limit}}$$

(22) $$\log\left(\frac{N}{N_{0}}\right)=-\frac{{\;k^{\prime }\;C}_{{\mathrm{Ag}}_{0}^{+}}}{\ln\left(10\right)}t+\frac{b}{\ln\left(10\right)}\;\mathrm{for}C_{{\mathrm{Ag}}^{+}}\geq C_{\mathrm{limit}}$$

where

k and k’ (L mg^−1^ min^−1^) are kinetic constants at 37 °C for the Ag^+^ antibacterial action;
C_limit_ (mol L^−1^) is a concentration limiting the first and the third zones ($C_{\mathrm{limit}}$ ≈ 0.3 mg L^−1^);
b (-) is an integration constant.

**Figure 7:**
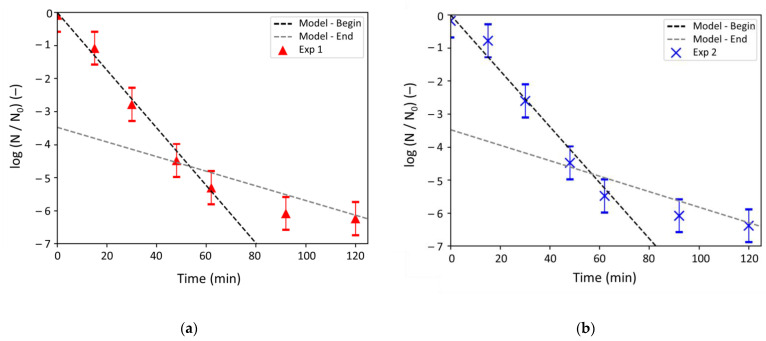
Log-removal values over time: model vs. experiment for (**a**) experiment 1 and (**b**) experiment 2. Model-Begin designates the model proposed over the first 60 min of experience while Model-End refers to the one from 60 min.

Table 4 gives the parameters of the Haas and Karra model determined by fitting the linear laws corresponding to Equations (21) and (22) with the respective linear regressions observed for the experiments. The fitting is showed in Figure 7.

A mean value of 6.60 ± 0.10 × 10^-2^ L mg^-1^ min^-1^ was thus obtained for k and 1.75 ± 0.05 × 10^-2^ L mg^-1^ min^-1^ for k’. Figure 7 shows the adequacy between the experiments and the Haas and Karra model, with both regression coefficients R^2^ and *χ^2^_reduce_* close to 1 (Table 4). To the best of our knowledge, the implementation of the Haas and Karra model to describe the biocidal action of silver ions is innovative, and was not reported yet in the literature.

### 2.4. Coupling of Silver Diffusion, Bacterial Growth, and Silver Antibacterial Action

Modeling and dissociated experiments have allowed determining the different kinetics of pure silver diffusion, bacterial growth, and biocidal action. In the next section, these kinetics will be combined to propose a global model simulating inhibition zones radii that will be confronted with experimental measurements.

#### 2.4.1. Model

The kinetics, thus, were coupled to simulate both diffusion and action of silver ions in the bacterial biofilm forming on the LB plate. For the simulation, two species were defined: silver ions (Ag^+^) and *E. coli* bacteria. Bacteria were admitted to grow on the agar only since the LB agar gel used is too viscous for bacteria diffusion inside the agar. As determined in Section 2.2, the bacterial growth rate was fixed to 0.78 h^−1^. Like silver diffusion within LB agar (Section 2.1.1), Ag^+^ ions were supposed to diffuse in the biofilm according to Fick’s Law (Equation (3)) with an unknown diffusivity D_Ag+ biofilm_. Bacteria were assumed to react with silver ions following the Haas and Karra kinetics defined in Section 2.3.3. Equation (20) was retained since inhibition halos were measured after at least 6 h of experiment and over 24 h in total.

The global bacterial rate resulted from two reactions (i.e., bacteria growth and biocidal action caused by silver ions) and was defined by Equation (23):

(23) $$\frac{\mathrm{dN}}{\mathrm{dt}}=\mu_{\max}N-\;k^{\prime }|C_{{\mathrm{Ag}}_{0}^{+}}\;N$$

where:

C_Ag0^+^_ is the initial silver concentration, ranging from 2 to 155 mg L^−1^ (i.e., from 0.19 to 1.44 mol m^−3^); testing different initial silver concentrations aimed to assess the robustness of the model;
N is the bacterial concentration expressed in the same unit as C_Ag0_^+^ (i.e., mg L^−1^ or mol m^−3^);
t (min) is the time;
k’ was fixed to 1.75 ± 0.05 L mg^−1^ min^−1^ (according to Section 2.3.3);
µ_max_ was fixed to 0.78 ± 0.06 h^−1^ (according to Section 2.2).

**Figure 8:**
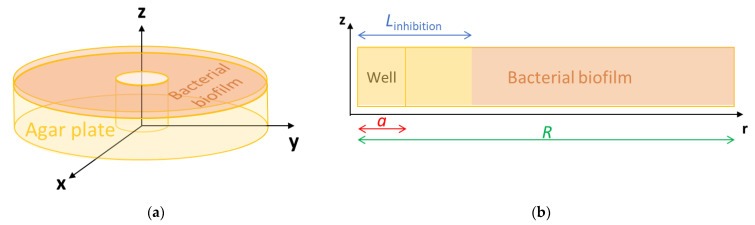
COMSOL geometry: (**a**) for the whole plate and (**b**) localized to the biofilm only.

These pieces of information were entered in the Chemical Reaction Engineering module of COMSOL. The mass transfer module was also completed to consider the diffusion kinetics, i.e., with the initial bacterial concentration, the density, viscosity, and thickness of the biofilm formed and the diffusivity D_Ag+ biofilm_ of the silver ions in the biofilm. The density of an *E. coli* biofilm made of living cells in an aqueous media was found to be 1.1 g mL^−1^ [33], while the dynamic viscosity was taken equal to 0.1 Pa s since this data were reported for a Gram-negative bacterium after 24 h incubation [34]. The initial bacterial concentration and biofilm thickness were determined on the basis of experimental results (detailed in Section 2.4.2). The diffusivity D_Ag+ biofilm_, i.e., the only unknown parameter, was assessed by fitting the model with the experiment to obtain similar bacterial inhibition. Figure 8 shows the geometry that was retained for the COMSOL simulation. It was the same as the cylindrical geometry used for pure diffusion (Section 2.1.1, Figure 1), which explains that initial and bound conditions were similar. Contrary to pure diffusion, the modeling was however focused on the biofilm only.

The implementation in COMSOL of the global model combining the reaction and diffusion kinetics was first intended to predict the respective concentration profiles of Ag^+^ (C_Ag+_) and *E. coli* (*N*) in the biofilm formed on the plate over time and space. The radius of the resulting inhibition halo (L_inhibition_) can be then assessed by considering the inhibition halo as the zone where the *E. coli* concentration was zero.

Fitting the global model (coupling all the kinetics involved) with the experiment to obtain similar inhibition radii should allow determining the D_Ag+ biofilm_ value. Depending on this value, two behaviors could be possible:

- Diffusion is the limiting phenomenon and a slight variation of D_Ag+ biofilm_ would cause strong shift between simulated and experimental results;
- Diffusion is not the limiting phenomenon and the simulation results would not be very sensitive to the D_Ag+ biofilm_ value (over a consistent range).

#### 2.4.2. Experiments

**Figure 9:**
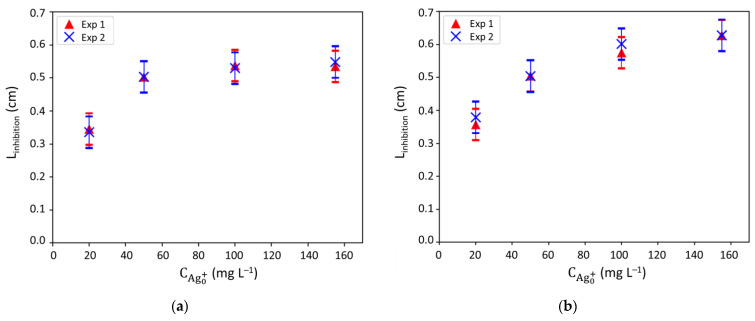
Inhibition halos radii (L_inhibition_) measured after 24 h of contact with silver ions (Ag^+^) on: (**a**) LB agar and (**b**) agar with similar composition of LB except that NaCl was removed; different initial concentrations of Ag^+^ were tested and each condition was replicated (Experiment 1 and Experiment 2, named Exp 1 and Exp 2, respectively).

Experiments were carried out as described in Section 3.3.4. The radius of the inhibition halo (L_inhibition_) was measured over time (Figure 9). Figure S2 illustrates the plates obtained for two initial silver concentrations tested (20 and 155 mg L^−1^) after 24 h incubation. As expected, the biofilm was only visible on the surface of the agar whatever the tested conditions. The measures of the inhibition halo radii were first done at 24 h to compare the influence of the presence of NaCl in the agar culture medium (Figure 9). Thus, two agar media were implemented for these tests: usual LB medium (Figure 9a) and medium with similar composition than LB, except for NaCl, was removed (Figure 9b).

Results were reproducible and evidenced in both cases that a minimal silver concentration of 20 mg L^−1^ was required to observe an action of silver against bacteria over 24 h (Figure 9). However, the liquid tests carried out to determine the antibacterial activity of Ag^+^ (Section 2.3.2 and Section 3.3.3) showed that a concentration of 3 mg L^−1^ was already sufficient. One hypothesis was that the presence of chlorides in the LB medium (that were not present in the phosphate buffer used for the liquid tests) could lead to Ag^+^ precipitation into AgCl and decrease the antibacterial activity of Ag^+^ [1]. Nevertheless, similar results were obtained for LB medium and LB medium exempt of NaCl (Figure 9a,b). Thus, chlorides were not responsible for the shift and results obtained on LB agar only will be presented in the rest of the study. Another explanation can be that the mass transfer of silver ions to bacteria was not limited in the liquid tests (where convection occurred), but limited in the biofilm formed on the agar. Therefore, these results suggest that diffusion could be the limiting phenomenon. This assumption is consistent with the fact that the inhibition halos radii were quasi similar from 100 to 155 mg L^−1^ (Figure 9).

**Figure 10:**
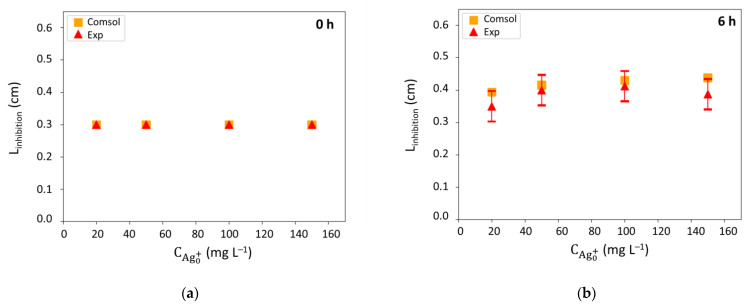

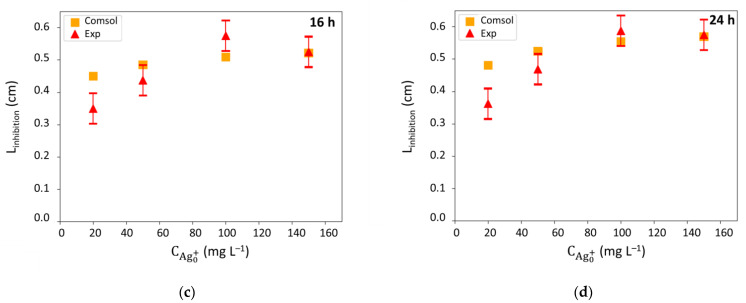
Inhibition halos radii (L_inhibition_): comparison between experimental (Exp) and model (Comsol) results at (**a**) 0 h, (**b**) 6 h, (**c**) 16 h, and (**d**) 24 h.

The inhibition zones radii (L_inhibition_) were then measured on LB agar medium after 6 h, 16 h and 24 h of incubation (Figure 10). Diffusion radii clearly increase with time and concentration. The inhibition zones reached a steady state after 6 h for the lowest silver concentration (20 mg mL^−1^) and after 16 h for the rest of the concentrations. It is worth noticing that no significant differences were observed from 100 and 150 mg mL^−1^.

Other experimental results are the initial bacterial concentration and the bacteria quantity forming the biofilm after 24 h incubation. The initial concentration of the bacteria spotted on the plate was 1.0 ± 0.2 × 10^9^ CFU mL^−1^, which corresponds to a mean mass concentration of 3.0 × 10^−4^ g_dried cell_.mL^−1^ (knowing that the mean weight of one dried bacteria is about 3 × 10^−13^ g, [25]) and finally to a mean molar concentration of 1.36 × 10^−2^ mol m^−3^ (since the average molar mass of a bacteria is 22 ± 12 kDa, [25]). Besides, the enumeration of bacteria in the biofilm recovered after 24 h incubation was found to be 3.2 ± 0.5 × 10^10^ CFU. According to literature, the bacterial density in the biofilm is about 3.6 × 10^10^ CFU mL^−1^ [33]. Thus, it was possible to determine the volume of the biofilm after 24 h (i.e., 3.2 × 10^10^ CFU/3.6 × 10^10^ CFU mL^−1^ = 0.9 mL). Knowing that the surface of the agar plate was 62 cm^2^, it comes a biofilm thickness around 145 µm, which is in accordance with literature [33].

#### 2.4.3. Comparison between the Model and the Experiments

**Figure 11:**
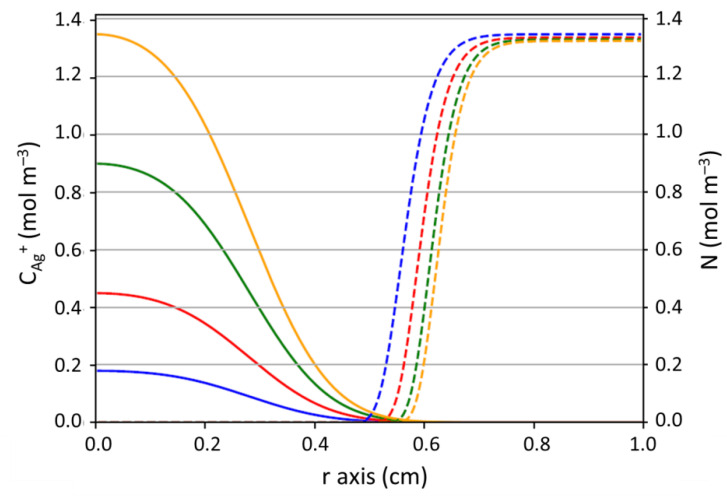
Concentration profiles after 24 h of contact (full lines: Ag^+^, i.e., C_Ag_^+^; dotted lines: *E.coli* bacteria, i.e., N). Blue curve: C_Ag0_^+^ = 20 mg L^−1^; red curve: C_Ag0_^+^ = 50 mg L^−1^; green curve: C_Ag0_^+^ = 100 mg L^−1^; yellow curve: C_Ag0_^+^ = 150 mg L^−1^.

The COMSOL simulation gave access to the concentration profiles for Ag^+^ (C_Ag_^+^) and *E. coli* (N) in the biofilm formed on the agar. These profiles are available over time and over space (along r axis). An overview of these spatial distributions after 24 h of contact is given in Figure 11.

The radius of the inhibition zone was defined as the smallest distance from the well center for which the *E. coli* concentration (N) was zero. After 24 h, Figure 11 shows that the bacterial concentration was zero up to a radius about 0.5 cm. In order to determine with precision the inhibition halos radii from the concentration profile of bacteria (Figure 11), a minimum of the curve giving N with time t (and thus with the r axis) was searched according to Equation (24). The solving of Equation (24) gave a silver concentration, which allowed to precise the radial distance since the profile of silver concentration was known with time and space.

(24) $$\frac{\mathrm{dN}}{\mathrm{dt}}=0\;\Leftrightarrow \;(\mu_{\max}-\;k^{\prime }|C_{{\mathrm{Ag}}^{+}})\;N=0\;\Leftrightarrow {\;C}_{{\mathrm{Ag}}^{+}}=\frac{\mu_{\max}}{k^{\prime }}$$

Different values of D_Ag+ biofilm_ were tested to fit the COMSOL simulations with the experimental results and a value of 7.0 ± 0.1 × 10^−12^ m^2^ s^−1^ was selected for the simulations. Results, as a function of time and silver concentrations, are presented in Figure 10.

A good correspondence between experimental results and simulation (Figure 10) tend to validate the model over 24 h for the highest concentrations (i.e., from 50 to 150 mg L^−1^) since the difference observed were included in the experimental error. Only the radii obtained for 20 mg L^−1^ show a significant difference. This can be explained by (i) the fact that the Minimum Inhibitory Concentration (MIC) of silver to observe inhibition is closed to this concentration, around which discontinuity in the inhibition size could be experimentally evidenced, which is not taken into account by the model; (ii) the small size of the experimental halo (Figure S2) inducing imprecision on the measures. The value of D_Ag+ biofilm_ (7.0 ± 0.1 × 10^−12^ m^2^ s^−1^) is nearly 400-times lower than the silver diffusivity in the agar (D_Ag+_). This is consistent with the presence of bacteria that hinder the silver transport. In addition, the predictions were much affected by slight variations on the value of D_Ag+ biofilm_ in the range tested (data not shown), which confirms the limitation by silver diffusion.

## 3. Materials and Methods

### 3.1. Materials

Solutions of silver ions (Ag^+^) were prepared by dissolving AgNO_3_ (Sigma, France) in deionized water. A red food dye (E122, Vahiné, France) was used when the silver solution staining was tested. Phosphate buffer (12.9 mM, pH 7.0 ± 0.1) contained KH_2_PO_4_ 1.06 g L^−1^ and Na_2_HPO_4_.12H_2_O 4.34 g L^−1^ (Sigma, France) and was prepared in deionized water. A non-pathogenic Gram-negative *Escherichia coli* bacterium (K12 DSM 423, from DSMZ, Germany) was used for the antibacterial study. For the bacterial culture, a ready-to use Lysogeny Broth (LB) Miller medium (Sigma, France) was implemented. LB medium contained peptone (10 g L^−1^), yeast extract (5 g L^−1^) and NaCl (10 g L^−1^) and was mixed with microbiologic agar (Sigma, France) at 15 g L^−1^ to obtain nutritive LB agar. Plates of 9 cm diameter were used. Each plate was filled with 25 mL of LB agar resulting in an agar thickness of 5 ± 1 mm. For all plates, an agar cylinder was sterilely removed from the plate center to form a well of 3.0 ± 0.4 mm radius and 5 ± 1 mm height. Before experiments, plates were pictured and the well diameters were precisely measured by using ImageJ program. Each well was filled with 50 µL of the silver solution (either dyed or not). Image J was also used to monitor the halos evolution over time.

### 3.2. Mathematical Modeling Software

For pure diffusion and phenomena coupling, modeling was carried out by COMSOL Multiphysics^®^ software (version 5.5). For mass transfer, the COMSOL module named Transport of diluted species was implemented. COMSOL uses the finite element method to solve differential equations. The model geometry and the mesh generation of the agar plate were chosen according to Chandrasekar et al. (2015) [20]. COMSOL data were then plotted with Python (version 3.7). For bacterial growth and silver biocidal action, Python (version 3.7) was used directly to implement the models.

### 3.3. Experiments

#### 3.3.1. Pure Diffusion of the Silver Ions in the Agar

A solution of silver ions (Ag^+^) was prepared at 100 mg L^−1^ then dyed with the red food dye E122 (5 mL of dye per 100 mL of silver solution). The stained silver solution was then put in the plate wells. In this experiment, plates were not inoculated by *E. coli* to decouple bacterial growth and inhibition from silver diffusion in the agar. Plates were thereafter incubated at 37 °C in aerobic conditions and pictured over time to monitor the formation and expanding of the dyed halos. The position of the camera compared to the plate was always the same. Images were then treated with the ImageJ software to measure the dyed halos sizes. The experiment was reproduced twice.

#### 3.3.2. Pure *E. coli* Bacterial Growth

Cultures were carried out from frozen *E. coli* aliquots stored at −20 °C in glycerol. Bacteria on the plates were expected to grow exponentially since they were pre-cultured just before agar inoculation. The growth rate of the bacterial culture was thus determined in the exponential phase on LB medium. In a 250 mL Erlenmeyer flask, LB medium (50 mL) was inoculated by an *E. coli* aliquot (10% *v/v*) and incubated on a rotary shaker (37 °C and 110 rpm) for 3 h. The flask was closed by a breathable cap ensuring sterility and continuous air entrance. Samples (1 mL) were withdrawn sterilely every 20–40 min and their optical density at 600 nm (OD_600_) was immediately measured. The optical density was correlated to the bacterial concentration in CFU mL^−1^ (where CFU means the colonies forming units, i.e., the bacteria number) by a correlation previously established [35]. The total volume of the samples taken during the kinetics did not exceed 10% of the total volume of the flask.

#### 3.3.3. Pure Antibacterial Action of Silver Ions

A liquid test was designed to avoid any transfer limitation of silver ions to bacteria. For each experiment, bacteria were first cultured. Cultures were carried out from frozen *E. coli* aliquots stored at −20 °C in glycerol. For that, LB medium was inoculated by an *E. coli* aliquot (4% *v/v*) and incubated overnight in aerobic conditions on a rotary shaker (30 °C and 110 rpm) up to reach the stationary phase and good physiological conditions for bacteria. The *E. coli* culture was then centrifuged (for 10 min at 4000 rpm, 12 °C) to separate the grown *E. coli* cells from the medium. The recovered *E. coli* pellets were thereafter re-suspended in phosphate buffer to stop further bacterial growth. A resting bacterial suspension, i.e., a suspension with non-growing bacteria, was indeed chosen to decouple bacterial growth from antibacterial action. The bacterial cells were diluted in phosphate buffer to reach an optical density at 600 nm (OD_600_) of 1.3 ± 0.1 and a sample of the suspension was taken to enumerate the bacterial concentration.

The bacterial suspension obtained was distributed in 25 mL Erlenmeyer flasks (16 mL per flask) closed by a breathable caps. Silver ions were then added to the bacterial suspension at a final concentration of 3.0 ± 0.1 mg L^−1^ (by adding 19.3 µL of a silver solution at 155 mg L^−1^ per mL of bacterial suspension). Flasks were then incubated on a rotary shaker (37 °C and 110 rpm) for 2 h and samples (100 µL) were withdrawn every 15 to 30 min for bacterial counting. The flask rotation allows convection, which induces non-limiting transfer between Ag^+^ and bacteria. Experiments were duplicated. A control reactor (bacteria without Ag^+^) was run simultaneously.

The bacterial concentrations (N, CFU mL^−1^) in the liquid samples were measured by the conventional plaque assay method. For that, liquid samples were diluted by decades and each dilution (50 µL) was spread onto LB agar plates. All plates were then incubated for 24 h at 37 °C. Once the cultivable bacteria had grown on plates, the colonies were counted on the plates where the dilution was appropriate, knowing that each colony stemmed from one initial bacterium. The concentrations N of bacteria in the samples were calculated as the average number of the colonies divided by the volume inoculated. The quantification limit was 20 CFU mL^−1^. Negative controls (i.e., without bacteria) were always run in parallel to check the sterility.

#### 3.3.4. Coupling of Silver Diffusion, Bacterial Growth and Silver Antibacterial Action 

The protocol carried out for this test is similar to the one described in Section 3.3.1, except that (i) plates were prior seeded by an *E. coli* culture before depositing the silver solution in the well, and (ii) the silver solution was not dyed. The *E. coli* culture was prepared by inoculating LB medium by an *E. coli* aliquot (4% *v/v*) and incubating the medium overnight on a rotary shaker (at 30 °C and 110 rpm) in aerobic conditions until the stationary phase was reached (that corresponds to an *E. coli* concentration of about 10^9^ CFU mL^−1^). Then 200 µL of the culture were spread on each LB agar plate. Immediately after, 50 µL of an Ag^+^ solution (at a concentration ranging from 2 to 155 mg L^−1^) was inserted in the well in the center of the agar plate. The plates were finally incubated for 24 h at 37 °C in aerobic conditions and pictured over time (at 6 h, 16 h, and 24 h). The position of the camera compared to the plate was always the same. Images were then treated with the ImageJ software to measure the inhibition halos sizes. The experiment was reproduced twice. A positive control, i.e., where silver solution was replaced by deionized water, was simultaneously carried out in the same conditions. After 24 h at 37 °C, the biofilm formed on the agar was wholly scratched and recovered in a known volume of phosphate buffer for re-suspension and enumeration by the plaque assay technique (detailed in Section 3.3.3). The bacteria quantity contained in the biofilm formed was thus determined by the knowledge of the bacterial concentration of the suspension obtained and its volume.

## 4. Conclusions and Outlooks

This study proposes an original approach and modeling to determine the diffusivity of silver ions in a bacterial biofilm and to fully characterize each phenomenon occurring during the bacteriostatic action of silver ions in an agar diffusion test. Experiments were implemented to determine kinetic constants regarding bacterial growth and silver action. COMSOL was used to simulate the dynamic coupling between bacterial growth, antimicrobial action, and silver diffusion. The fitting between experiments and model allowed to determine the researched diffusivity and demonstrated the mass transfer limitation when silver concentrations were higher than MIC. The study also resulted in a global model allowing the simulation of the bacteriostatic system as function of time and space for different silver concentrations. Recent studies show the interest of simulation to solve and overpass experimental problems [20]. The present protocol using both experimental results and model could be extended to simulate other bacteriostatic systems, such as antibacterial surfaces for biomedical or food applications (plasters, membranes, or packaging).

## Figures and Tables

**Table 1:** Optical density at 600 nm (OD_600_) measured over time for the bacterial culture during the exponential growth phase. Four independent experiments were done (Exp 1, Exp 2, Exp 3 and Exp 4 respectively).

**Exp 1**	time (h)	0.37	1.0	1.65	2.33
	OD_600_ (-)	0.31	0.56	1.04	1.51
**Exp 2**	time (h)	0.32	1.02	1.63	2.32
	OD_600_ (-)	0.31	0.58	1.13	1.48
**Exp 3**	time (h)	0.45	0.92	1.92	2.43
	OD_600_ (-)	0.30	0.49	1.10	1.46
**Exp 4**	time (h)	0.5	1	1.5	2
	OD_600_ (-)	0.40	0.60	0.80	1.15

**Table 2:** Values obtained for the maximal growth rate (µ_max_).

Exp	µ_max_ (h^−1^)	R^2^ (-)
1	0.83	0.9847
2	0.81	0.9684
3	0.80	0.9901
4	0.69	0.9961
**Mean value**	0.78	
**Standard deviation**	0.06	

**Table 3:** Bacterial concentration N (colonies forming units (CFU) mL^−1^) measured over time for two experiment replicates (Exp 1 and Exp 2, respectively). A control reactor without Ag^+^ ions was carried out simultaneously.

Time (min)	0	15	30	48	62	92	120
Control (CFU mL^−1^)	7.5 × 10^8^	7.5 × 10^8^	1.6 × 10^9^	8.8 × 10^8^	1.2 × 10^9^	1.1 × 10^9^	1.5 × 10^9^
Exp 1 (CFU mL^−1^)	6.3 × 10^8^	6.3 × 10^7^	1.3 × 10^6^	2.5 × 10^4^	3.8 × 10^3^	6.3 × 10^2^	4.4 × 10^2^
Exp 2 (CFU mL^−1^)	5.0 × 10^8^	1.3 × 10^8^	1.9 × 10^6^	2.5 × 10^4^	2.5 × 10^3^	6.3 × 10^2^	3.1 × 10^2^

**Table 4:** Values obtained for the parameters of the Haas and Karra model from experiments (Exp 1 and Exp 2, respectively).

Exp 1			Exp 2		
*For C_Ag+_ ≤ C_limit_*			*For C_Ag+_ ≤ C_limit_*		
	**Value**	**Standard deviation**		**Value**	**Standard deviation**
*k* (L mg^−1^ min^−1^)	6.7 × 10^−2^	0.8 × 10^−2^	*k* (L mg^−1^ min^−1^)	6.5 × 10^−2^	0.8 × 10^−2^
R^2^ (-) *	0.9873		R^2^ (-)	0.9383	
χ^2^_reduce_ **	1.22		*χ^2^_reduce_*	1.05	
*For C_Ag+_ ≥ C_limit_*			*For C_Ag+_ ≥ C_limit_*		
	**Value**	**Standard deviation**		**Value**	**Standard deviation**
*k’* (L mg^−1^ min^−1^)	1.7 × 10^−2^	0.2 × 10^−2^	*k’* (L mg^−1^ min^−1^)	1.8 × 10^−2^	0.2 × 10^−2^
*b* (-)	−8	5	*b* (-)	−8	5
R^2^ (-)	0.9834		R^2^ (-)	0.9907	
*χ^2^_reduce_*	0.99		*χ^2^_reduce_*	1.17	

* R^2^ is the regression coefficient. ** χ^2^_reduce_ is defined as the sum of the ratios squared of the residues to the measurement uncertainty, divided by the number of points considered.

## Data Availability

Data is contained within the article.

## Supplementary Materials

The following are available online at https://www.mdpi.com/article/10.3390/antibiotics10040368/s1, Figure S1: Evolution of dyed halos over time: (a) after 60 min and (b) after 155 min experiment. Each plate was duplicated. Figure S2. Pictures of inoculated plates in contact with silver ions for 24 h; initial silver concentrations were respectively (a) 20 mg L^−1^ and (b) 155 mg L^−1^.
