# Supplementary material for: Mechanistic Study of the Kinetic Phenomena Influencing the Bacteriostatic Action of Silver Ions in Agar Bioassays
Source: Antibiotics (Basel). 2021 Mar 31;10(4):368. doi: 10.3390/antibiotics10040368 (PMC8065754; doi:10.3390/antibiotics10040368)
Supplement: Supplementary file 1 [file antibiotics-10-00368-s001.pdf]

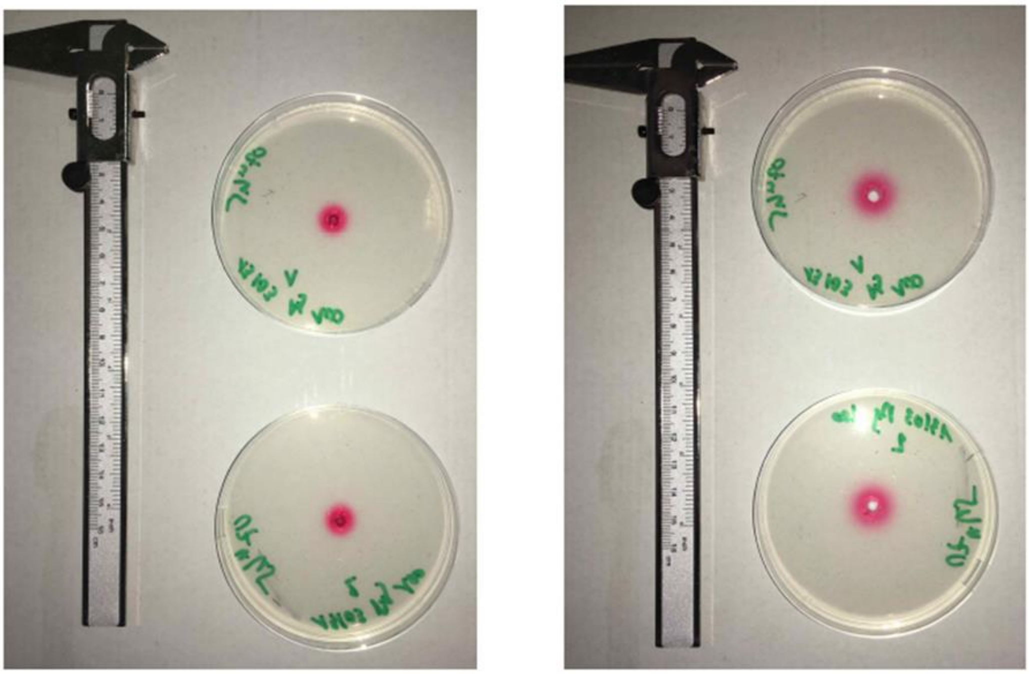

(**a**) (**b**)

Figure S1. Evolution of dyed halos over time: (**a**) after 60 min and (**b**) after 155 min experiment. Each plate was duplicated.

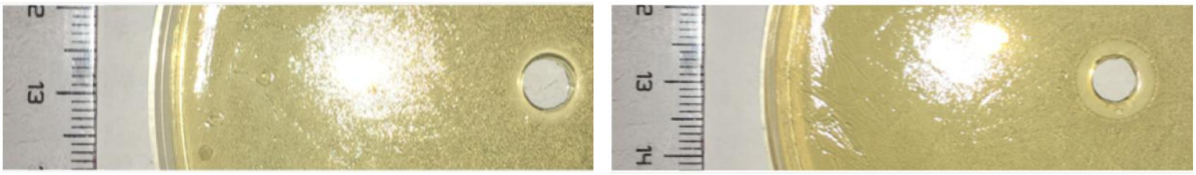

(**a**) (**b**)

Figure S2. Pictures of inoculated plates in contact with silver ions for 24 h; initial silver concentrations were respectively (**a**) 20 mg L^{-1} and (**b**) 155 mg $L^{-1.}$
